# Alteration of the Intra- and Cross- Hemisphere Posterior Default Mode Network in Frontal Lobe Glioma Patients

**DOI:** 10.1038/srep26972

**Published:** 2016-06-01

**Authors:** Haosu Zhang, Yonghong Shi, Chengjun Yao, Weijun Tang, Demin Yao, Chenxi Zhang, Manning Wang, Jinsong Wu, Zhijian Song

**Affiliations:** 1 Neurosurgery Department, Huashan Hospital, Shanghai Medical College, Fudan University, China; 2 Digital Medical Research Center, School of Basic Medical Sciences, Fudan University, China; 3 Shanghai Key Laboratory of Medical Imaging Computing and Computer-Assisted Intervention, 200032 Shanghai, China.

## Abstract

Patients with frontal lobe gliomas often experience neurocognitive dysfunctions before surgery, which affects the default mode network (DMN) to different degrees. This study quantitatively analyzed this effect from the perspective of cerebral hemispheric functional connectivity (FC). We collected resting-state fMRI data from 20 frontal lobe glioma patients before treatment and 20 healthy controls. All of the patients and controls were right-handed. After pre-processing the images, FC maps were built from the seed defined in the left or right posterior cingulate cortex (PCC) to the target regions determined in the left or right temporal-parietal junction (TPJ), respectively. The intra- and cross-group statistical calculations of FC strength were compared. The conclusions were as follows: (1) the intra-hemisphere FC strength values between the PCC and TPJ on the left and right were decreased in patients compared with controls; and (2) the correlation coefficients between the FC pairs in the patients were increased compared with the corresponding controls. When all of the patients were grouped by their tumor’s hemispheric location, (3) the FC of the subgroups showed that the dominant hemisphere was vulnerable to glioma, and (4) the FC in the dominant hemisphere showed a significant correlation with WHO grade.

Approximately 15,750 new cases of malignant brain gliomas are diagnosed in adults in the United States each year^1^. A malignant glioma originates from the glial tissues, grows in the parenchyma and eventually infiltrates cerebral functional regions due to the degradation of the extracellular matrix and adhesion molecules. Gliomas can cause a variety of symptoms, including headaches, nausea, vomiting, seizures, cranial nerve disorders, and visual loss. For the noninvasive assessment of the microscopic infiltration of tumors, functional magnetic resonance imaging (fMRI) has emerged as an advanced tool for detection and diagnosis because resting-state fMRI can be used to analyze the brain’s functional state by evaluating the neurological integrity of cognitive networks.

The resting-state default mode network (DMN) is the most easily detected and consistent cluster; it consists of a set of areas encompassing the posterior cingulate cortex (PCC), anterior cingulate cortex (ACC) and temporal parietal junction (TPJ) and shows more activity at rest than other clusters^2^. The reproducibility of the network across patients and studies makes it a target of interest in functional connectivity research. Numerous studies have shown alterations of the DMN in neurological conditions such as Alzheimer’s disease^3^, schizophrenia^4^, and dementia^5^.

The exact functional roles of the DMN in frontal lobe tumors remain unclear. The existing studies of these functional roles are generally classified into the anterior, posterior, left, and right areas of the DMN^6^,^7^,^8^,^9^,^10^. Most studies acknowledge that gliomas may cause alterations of DMN connectivity to varying degrees due to their different locations and grades of deterioration^11^,^12^,^13^,^14^,^15^,^16^. For example, with brain tumor growth, Harris *et al*. found that higher-grade tumors along with prior surgery and/or treatment cause larger reductions in DMN functional connectivity (FC) in patients with primary gliomas, and the location of tumors inside and/or outside DMN regions has an impact on FC^17^. However, most of the available research was focused on only unilateral gliomas, without simultaneously considering hemispheric differences, until a study by Buklina *et al*. who concluded that the different degrees of damage to language functions caused by hemispheric tumors are directly related to hemispheric differences^16^. This finding suggests that hemispheric differences in the DMN should be taken into consideration in the presence of a glioma. At the same time, Wu *et al*. discovered that the functional strength of tumor regions is related to their World Health Organization (WHO) grade^18^. Motivated by these studies, the present study mainly concentrated on the alterations in FC strength in the posterior DMN, intra- and cross-hemispherically, in unilateral frontal lobe glioma patients.

Specifically, regions selected from the PCC and TPJ areas of the posterior DMN were used to build seed-based functional brain maps to determine the changes in the hemispheric difference. Here, a brain map was built from the seed regions located in the left and right PCC to the target regions determined in the left and right TPJ to observe the strength of FC, which reflects inner functional changes as the anterior area of the DMN is accompanied or invaded by a frontal glioma. We hypothesized that the changes in the pattern of FC strength intra- or cross-hemispherically would not occur at the same level and would exhibit different sensitivities to a frontal tumor. Specifically, we focused on the following questions in this study: (*i*) Does the seed-based DMN accompanying a unilateral frontal tumor exhibit a hemispheric difference? (*ii*) What is the degree of this difference? (*iii*) How strong is the correlation between the intra- or cross-hemisphere difference and WHO grade? To infer these signal coherencies, we applied a region-of-interest (ROI)-based correlation analysis approach to estimate the spatial characteristics of the resting-state signal fluctuations.

## Methods

### Subjects and Assessment

A total of 20 patients with histologically confirmed gliomas and 20 healthy control volunteers (HCs) were included in the study. No medicine was taken before fMRI scanning. Anyone with a history of drug or alcohol addiction, cerebrovascular disease or mental disease was excluded. All processes strictly followed the requirements of the Declaration of Helsinki. This study was approved and supervised by the Ethics Committees of Huashan Hospital. After a clear explanation of the research, written informed consent was obtained from the healthy volunteers and the patients’ legal guardians. The included subjects had no history of drug or alcohol addiction, cerebrovascular or mental disease, or congenital brain disease (i.e., congenital hydrocephalus).

Patient data from 2010 to 2014 included in our institutional database were retrospectively scanned for pre-surgical planning. The patients were screened to include individuals with hemispheric frontal lobe gliomas and without impairments in posterior DMN areas, as confirmed during surgery and through pathological testing. Table 1 shows the demographics and clinical data of the 20 patients (11 men and 9 women, average age 44.25 years). The 20 HCs consisted of 13 men and 7 women with an average age of 33.55 years. All of the patients and HCs were right-handed according to the Edinburgh Handedness Inventory^19^. According to WHO grades, 10 patients had low-grade gliomas (WHO I & II), and 10 had high-grade gliomas (WHO III & IV). Furthermore, 12 of the patients had tumors located in the left frontal lobe and 8 in the right. Pathological diagnosis revealed that 8 patients had astrocytoma, 4 had glioblastoma, 3 had oligodendroglioma, 2 had anaplastic oligodendroglioma, and that anaplastic granular astrocytoma, glioma and anaplastic astrocytoma were each observed in 1 patient. The included subjects were not related to each other or to individuals conducting and directing the experiment.

### MRI Data Acquisition and Preprocessing

A Siemens Magnetom Verio 3.0 T MRI scaner was used to scan the patients in the Department of Radiology of Huashan Hospital, Shanghai, China. The structural scans were axially acquired using 3-dimensional T1-weighted magnetization-prepared rapid gradient echo (MPRAGE) with the following parameters: repeat time (TR) = 1900 ms; echo time (TE) = 2.93 ms; flip angle (FA) = 9°; matrix size = 256 × 215; field-of-view (FOV) = 250 mm × 218 mm; slice number = 176; slice thickness = 1 mm; acquisition averages = 1; and scanning time = 7 min 49 sec. Resting-state blood oxygenation level-dependent (BOLD) fMRIs were axially collected using an echo planar imaging (EPI) sequence, with the following parameters: TR = 2000 ms; TE = 35 ms; FA = 90°; matrix size = 64 × 64; FOV = 210 mm × 210 mm; slice number = 33; slice thickness = 4 mm; and gap = 4 mm. Each scan lasted 8 min and comprised the volumes of 240 time points per subject. During scanning, the subjects were instructed to remain as motionless as possible, keep their eyes closed and not fall asleep. If the images did not meet the radiological standards (i.e., improper position or abnormal shadow), a re-scan was performed at least 4 weeks later to guarantee inter-subject reliability over time^20^.

All MR imaging data were preprocessed with the AFNI tool (Analysis of Functional Neuroimages, http://afni.nimh.nih.gov/). A radiologist, two neuroscientists and two neurosurgeons with more than 5 years of experience were involved in data preprocessing to ensure the objectivity of the results.

After converting the DICOM data into AFNI-readable images, the volumes of the first 4 time points of the functional time series for each patient were truncated for magnetization equilibrium. The fMRI data were preprocessed by following the conventional five steps: ① Correction of head motion to obtain consistent anatomical coordinates^20^,^21^,^22^,^23^; i.e., images showing head motion with a displacement of greater than 0.5 mm or rotation of greater than 1.5 degrees in any direction throughout the course of scanning were excluded. ② Correction of slice timing to obtain a consistent acquisition time using the temporal derivative. ③ Spatial smoothing with a 6 mm isotropic Gaussian kernel to compensate for inter-subject gyral variability and attenuate high-frequency noise. ④ Temporal filtering to remove slow linear drifts to reserve signals between 0.01 Hz and 0.1 Hz, which is generally regarded as the main range of neural fluctuations^24^,^25^. ⑤ Global intensity normalization of all images to the Talaraich standard space of the Montreal Neurologic Institute template using the specified approaches (i.e., registration of the fMRI data to the corresponding T1 image, transformation of the anatomical image (i.e., T1 image) to match the template image and obtaining normalization parameters, which were then applied into the fMRI images for mapping to the template space).

**Figure 1:**
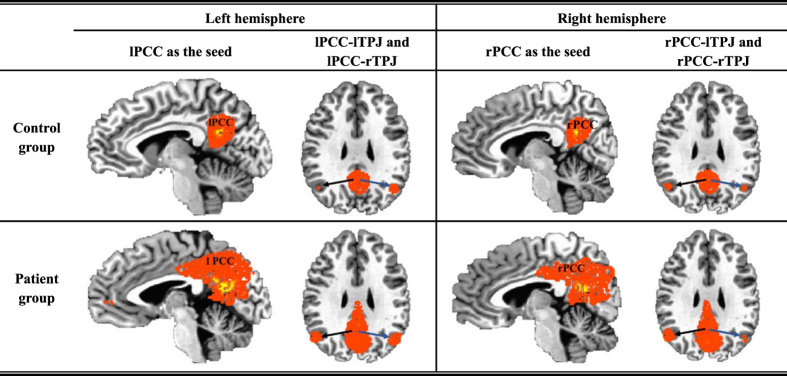
Illustration of the highlighted co-active regions in the PCCs and TPJs within the control and patient groups.

After the images were normalized to Talaraich standard space, with the help of the AFNI region-picking tool, we obtained the PCC regions of interest in the left and right hemispheres, which were regarded as the seeds of the left and right PCC and denoted by lPCC and rPCC, respectively. Then, the correlation coefficients between the seeds and other brain regions, such as the left or right TPJ, were calculated. A seed-based brain functional map was constructed for each individual subject and transformed to Fisher’s Z distribution for group-level T-tests^23^. Thus, the set of FC maps followed a normal distribution with a zero mean. Furthermore, the target regions were defined as spheres with a radius of 5 mm whose centers were the voxels with the highest Z score found in the left and right TPJ areas (denoted by lTPJ and rTPJ, respectively). Accordingly, the calculated hemispheric Z scores, which also represented the seed-based hemispheric FC strength, indicate the relationships of lPCC-lTPJ, lPCC-rTPJ, rPCC-lTPJ and rPCC-rTPJ; here, ‘l’ and ‘r’ indicate that the partitions of PCC and TPJ were in the left or right hemisphere, respectively. All of the FC strength values were input into IBM SPSS (version 20) for statistical analysis. Here, Fig. 1 showed the consistency of the selected anatomical strucures from patients and controls, respectively. The volume of the total voxels in each tumor lesion was also calculated via AFNI; the volume of the total voxels in normal tissues and the ratio between the tumor lesion and normal tissue were estimated and are listed in Table 1. The tumor regions were verified by neurosurgeons with more than 10 years of working experience.

### Statistical Analysis

**Figure 2:**
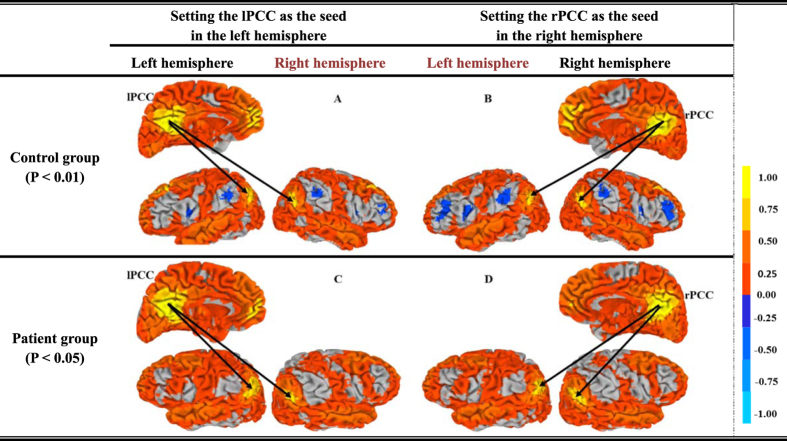
Illustration of the brain functional connectivity map based on the lPCC and rPCC seeds (yellow regions) based on a single-sample T test (P < 0.01 for the control group on the top line, and P < 0.05 for the patient group on the bottom line). Here, the lTPJ and rTPJ regions exhibit the strongest correlation with the PCCs and are displayed in dark yellow.

**Figure 3:**
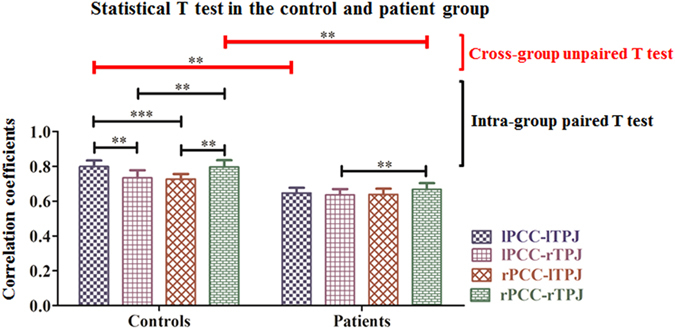
Illustration of the intra- and cross-group relationships among lPCC-lTPJ, lPCC-rTPJ, rPCC-lTPJ, and rPCC-rTPJ determined using a paired or unpaired T test (P < 0.05). (‘**’ indicates P < 0.05 and ‘***’ indicates P < 0.01). (See details in Charts 3A and 3B and Charts 4 and 5 in the Appendix).

**Figure 4:**
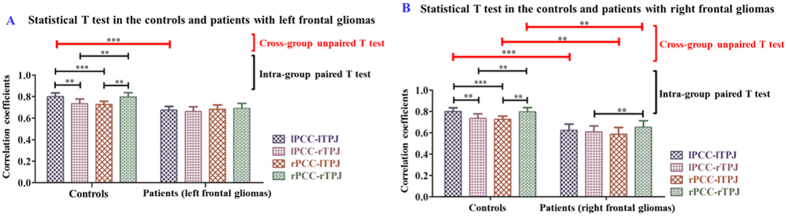
Illustration of paired and unpaired T tests calculated in the patient group with frontal gliomas in the left (**A**) or right (**B**) hemispheres (‘**’ indicates P < 0.05 and ‘***’ indicates P < 0.01). (See details in Charts 8–12 in the Appendix).

To determine whether a hemispheric difference existed in the patient and the control groups, Student’s T-test was used. A single-sample T-test was calculated to demonstrate intra-group consistency (i.e., within the patient and control groups) (Fig. 2, Table 2). A paired T-test was applied to evaluate the changes in intra-hemispheric FC strength between PCC and TPJ within groups (Figs 3 and 4, Table 3). An independent samples T-test was used to analyze the inter-group changes in FC strength (i.e., between the patients and controls) (Figs 3 and 4, Table 3). We also computed partial correlation coefficients describing the linear relationship between FC strength and WHO grade while regarding the volume of normal cerebral tissue and that of the tumor lesion as covariates (Tables 4 and 5). Additionally, the details of the statistical analysis are explicitly described to indicate the types of data to which the different T-tests were applied and are provided in the Appendix file.

## Experimental Results

### Analysis of the total data

For discovering the effect of the frontal glioma on the DMN, this study analyzed the cerebral hemispheric functional connectivity in the brain areas without the tumor’s invasion by means of the ROI-based method to pick up the brain regions from each subject. To illustrate the consistency of the selected anatomical strucures from patients and controls, we use a voxel-based multiplication to show the co-active regions among all subjects within each group. Here, 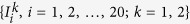 denote the subjects from the control (*k* = 1) or patient (*k* = 2) group, and 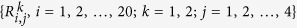 denote the regions of each subject within each group. Then, for the *m*^th^ voxel in the 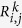 region, 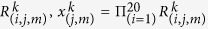 represents the co-active voxels from the corresponding regions from all subjects within group *k*. All of the 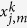 comprise co-active regions that still correspond to the highlighted PCC and TPJ regions shown in Fig. 1. We also found that the highlighted regions in the controls were more concentrated than those in the patients.

Then, for P < 0.01, Fig. 2(A,B) show the correlated regions that were estimated in the control group based on the lPCC and rPCC seeds, respectively. For P < 0.05, Fig. 2(C,D) show the corresponding correlated regions in the patient group. According to the color bar, the control group maintained intra-group consistency, with almost all regions being highly correlated with the lPCC or rPCC seeds displayed in yellow. Meanwhile, the lTPJ and rTPJ regions indicated in dark yellow showed the highest correlation with the PCCs in the brain maps, indicating that the PCC and TPJ regions were the most correlated among all brain regions, which is consistent with the DMN. Similarly, in the patient group, the correlations between the PCCs and TPJs were also very strong, despite the existence of frontal brain gliomas.

Table 2 also shows the FC strength values of the control and patient groups in the 95% confidence intervals (CI), which clearly illustrates the difference in the average FC strength between the controls and patients. From these figures and table, we can see that all of the FC strength values were lower in the patient group than in the control group because of the frontal lobe gliomas.

Furthermore, paired or unpaired T-tests were used to analyze the intra- and across-group relationships among lPCC-lTPJ, lPCC-rTPJ, rPCC-lTPJ, and rPCC-rTPJ. First, Levene’s test (P > 0.1) showed that all pairs exhibited homogeneity of variance. Second, as shown in Fig. 3, a paired T-test (P < 0.05) showed that there were significant differences between the intra- and cross-hemispheric FC in the control group, which confirms the results of a previous study^26^. We also found that the intra-hemispheric FC strength was higher than the cross-hemispheric strength in the control group. For the patient group, a significant difference was maintained for only the relationship of the right TPJ with the PCCs, whereas the remaining pairs (including the intra-hemispheric and cross-hemispheric pairs) showed no difference. Third, an unpaired T test (P < 0.05) between the patient and control groups demonstrated that the intra-hemispheric FC strength was significantly decreased, whereas no difference was detected in the cross-hemispheric pairs.

Table 3 shows the Pearson correlation coefficients calculated between the pairs of FC strength values within the groups. It can be observed that different PCC-TPJ pairs are significantly correlated not only in the control group but also in the patient group. Moreover, the correlation coefficients between the FC pairs in the patient group are higher than the ones in the control group due to the frontal gliomas.

### Analysis of sub-group data

Based on the location of the glioma in the hemisphere, we classified the total patient data into 2 sub-groups: one consisting of 12 left frontal glioma patients and the other of 8 right frontal glioma patients, and we then calculated the corresponding statistics. Figure 4(A) illustrates the statistical tests for the controls and the patients with left frontal glioma. First, Levene’s test (P > 0.1) demonstrated homogeneity of variance. Second, the unpaired T test (P < 0.05) showed that the patients with left frontal glioma suffered a significant decrease in left intra-hemispheric FC strength (i.e., the connectivity of lPCC-lTPJ). Third, for the patient group, paired T tests were also calculated for intra- and cross-hemisphere comparisons, but no difference was found. The intra-hemispheric FC strength was obviously greater than the cross-hemispheric FC strength, although no difference was detected. Figure 4(A) shows that there were no significant differences in the patient group with left frontal gliomas.

Similarly, Fig. 4(B) illustrates the statistical test in the controls and in the patients with right frontal gliomas. First, Levene’s test (P > 0.1) indicated homogeneity of variance. Second, the unpaired T test showed that the patients with right frontal gliomas suffered a significant decrease not only of the right intra- and cross-hemispheric FC strength (P < 0.05) but also of the left intra-hemispheric FC strength (P < 0.01). Third, a paired T test (P < 0.05) showed that the difference in the FC strength of the relationship of the right TPJ with the PCCs was maintained in the patient group.

Figure 4 shows that the left intra-hemisphere FC strength (i.e., lPCC-lTPJ) was significantly decreased in both groups, regardless of the hemispheric location of the frontal tumor. Compared with the control group, there was an intra-hemispheric difference in both the left frontal glioma group and the right frontal tumor group; a significant difference in the FC of lPCC-lTPJ, rPCC-lTPJ and rPCC-rTPJ can be observed.

### Relationship between functional connectivity and WHO grade

Generally, the pathological diagnosis of glioma is made according to the WHO grade. In the group of patients included in this study, 10 exhibited WHO grade II, 6 exhibited WHO III, and 4 exhibited WHO IV. Grades I-II are regarded as low-grade gliomas (LGGs), and grades III-IV are classified as high-grade gliomas (HGGs). Thus, 10 LGG and 10 HGG patients were included in this study. Correlation analysis of the total patient data revealed no significant difference between the WHO grades and any FC pair of PCC and TPJ.

Because it has previously been shown that functional differences exist between the two hemispheres, the sub-groups of the patients described above were further studied to determine the relationships with WHO grades through correlation analysis. Table 4 shows that the FC pair of lPCC-lTPJ in the dominant hemisphere presented a significant correlation with WHO grade, which confirms the results of previous studies to some extent^18^. Furthermore, the volumes of normal cerebral tissue and tumor lesion were set as covariates, and partial correlation analysis was used to observe the relationship between WHO grades and FC pairs. However, no significant results were found, as shown in Table 5. These results are in contrast to previous studies in which it was found that functional strength was related to WHO grade^17^,^18^. Here, we argue that a larger fMRI database of glioma patient data may be necessary for validation.

### Discussion and Conclusion

The DMN has been considered to reflect cerebral FC^27^. This study was designed to determine how gliomas in the frontal lobes affect the DMN by calculating the patterns of FC strength in the reserved posterior DMN (i.e., FC pairs of (r/l)PCC-(r/l)TPJ). In this process, hemispheric differences were paid close attention. The common way to detect changes in the DMN and the consciousness of patients with traumatic brain injury is through whole-brain functional mapping, which may include tumor regions and their peripheral areas. Previous studies have shown that the anatomical structures of these regions are damaged or exhibit astrocyte proliferation and repairment^28^,^29^; therefore, it is difficult to differentiate whether the origins of their signals are the preserved neurons. In patients with frontal gliomas, the comparably preserved posterior regions are better able to reflect changes in the DMN. To make the analysis more robust, it was ensured that there were no underlying structural lesions in the posterior DMN region via the experimental design. It is apparent that gliomas not only affect local cerebral function but also decrease the whole-brain functional state. Moreover, posterior brain regions without tumor infiltration also suffered a decrease in FC strength.

(*i*) In the control group, intra-hemispheric FC strength was stronger than cross-hemispheric FC strength, which was decreased or lost in the group of patients with a frontal glioma (Figs 1, 2, 3). The strength patterns of all FC pairs of PCC and TPJ (which are the regions belonging to the DMN) were highly correlated with the frontal glioma and changed in the same way. Specifically, the strength of the intra-hemispheric FC of the DMN (i.e., lPCC-lTPJ and rPCC-rTPJ) decreased from 0.799 ± 0.155 and 0.797 ± 0.169 in the controls to 0.647 ± 0.133 and 0.668 ± 0.159 in the patients with frontal glioma, respectively (Table 2). Meanwhile, the confidence intervals shown in Table 2 indicated that the seed-based FC patterns in the patients were affected by the frontal lesions, differing from those in the controls. (*ii*) Regardless of the side of the frontal glioma, the decrease in the strength of left intra-hemispheric FC (i.e., lPCC-lTPJ) indicated vulnerability of the dominant hemisphere, considering that the subjects were all right-handed (Fig. 4). The FC in the posterior DMN (e.g., PCCs-TPJs) could be affected by the tumor in the anterior lobe to different degrees, which reveals the existence of a difference in hemispheric sensitivity.

First, intra-group analysis of the controls indicated that the significant difference observed between intra- and cross-hemispheric FC strength was consistent with previous studies^30^,^31^. However, in the patient group, this difference was not detected. Second, the intra-hemispheric FC strength was reduced more than the cross-hemispheric FC strength in the patient group, which indicates that intra-hemispheric FC is more vulnerable. Additionally, it should be noted that surgery and pathological diagnosis confirmed the hemispheric locations, which supports the hemispheric lateralization of the DMN with glioma and is consistent with Karl’s viewpoints about hemispheric specialization^32^. Previous studies have demonstrated that a simple task tends to be handled intra-hemispherically, whereas a complex task shows bilateral hemisphere involvement^33^, which indicate a hemispheric difference. The TPJ regions are located in different hemispheres, and there is no anatomical evidence that they are connected by nerve fibers. In other words, the TPJ regions are isolated from each other, and their relationship with PCCs can reflect hemispheric differences. These characteristics provide a way of understanding the evolution of hemispheric asymmetries and the lateralization of their functions.

However, the intra-group correlation coefficients of the patients were higher than those of the controls (Table 3). It is possible that the circulatory balance was interrupted by the glioma’s blood supply. First, a recent study on glioma showed that the distinct tumor microtubes can serve as routes for brain invasion, proliferation, and interconnection over long distances^34^, providing anatomical evidence that the alteration of the posterior DMN reflects the interruption of the anterior DMN. Second, due to neovasculature around cellular structures, leading to high vascularity and metabolism of a glioma, there is increased blood flow in the tumor-related region, which relatively reduces the perfusion of the entire brain region^18^. Cebeci’s research confirmed that the relative cerebral blood flow (rCBF) of the cerebral blood supply is related to glioma grade^35^. Thus, consistent reductions in the nutrient supply to the PCC and TPJ regions leads to a stronger correlation between them. It can be concluded that glioma results in an overall decrease in cerebral perfusion, causes elimination of the hemispheric differences in the perfusion volume of various cerebral regions, and finally, influences blood oxygenation level-dependent signals. Glioma is a key clue to the dysfunctional cerebral state compared with the normal brain.

Furthermore, all of the subjects included in this study were dextro-manual, exhibiting a dominant left hemisphere. Our results also demonstrated functional alteration of hemispheric differences (Fig. 4). Because there were no structural deficits observed in the posterior DMN regions, the alterations observed here could be regarded as purely functional (rather than structural). The experimental results from the sub-group analysis showed that the dominant hemisphere was more sensitive than its non-dominant counterpart; however, validation of this finding will require additional data. The authors propose that differences in hemispheric perfusion may be responsible for this finding^36^.

Finally, when considering the total patient data, no significant correlation was observed between the functional connectivity of (r/l)PCC-(r/l)TPJ and WHO grade (Tables 4 and 5). When the subgroups were analyzed based on the location of the glioma, we found a significant correlation between the left intra-hemispheric FC and WHO grade, which we will further validate in a larger population sample in the future.

The properties of the DMN have been studied in healthy subjects and in patients with brain pathologies such as Alzheimer’s disease^3^, schizophrenia^4^ and depression^37^, and the findings have been translated into clinical practice. The FC of the posterior DMN may serve as a potentially useful biomarker of brain disease. We may also conclude that in rehabilitation treatment, hemispheric differences should be taken into consideration.

## Additional Information

**How to cite this article**: Zhang, H. *et al*. Alteration of the Intra- and Cross- Hemisphere Posterior Default Mode Network in Frontal Lobe Glioma Patients. *Sci. Rep.* **6**, 26972; doi: 10.1038/srep26972 (2016).

## Supplementary Material

Supplementary Information

## Figures and Tables

**Table 1:** Demographics and clinical data of the patients.

No.	WHO grade	Major frontal location	Sex	Age (years)	Pathological diagnosis	Number of voxels in normal tissue	Ratio of tumor volume to normal cerebral volume
1	II	Left superior	M	41	Astrocytoma	1,453,693	0.0239
2	II	Left inferior	F	58	Astrocytoma	1,481,566	0.0039
3	II	Left inferior	F	43	Astrocytoma	1,356,003	0.0452
4	II	Left superior	F	35	Astrocytoma	1,512,911	0.0145
5	II	Left inferior	M	39	Oligodendroglioma	1,396,611	0.0345
6	II	Left middle	F	57	Astrocytoma	1,364,875	0.0489
7	II	Left middle	F	40	Oligodendroglioma	1,461,697	0.0079
8	III	Left superior	F	27	Anaplastic granular astrocytoma	1,378,627	0.0528
9	III	Left inferior	M	45	Glioma	1,482,300	0.0352
10	IV	Left inferior	M	67	Glioblastoma	1,309,974	0.0635
11	IV	Left inferior	M	42	Glioblastoma	1,334,129	0.0463
12	IV	Left inferior	M	49	Glioblastoma	1,322,207	0.0899
13	II	Right middle	M	47	Oligodendroglioma	1,501,317	0.0146
14	II	Right inferior	M	45	Astrocytoma	1,382,652	0.0401
15	II	Right middle	F	51	Astrocytoma	1,420,806	0.0443
16	III	Right middle	M	57	Anaplastic oligodendroglioma	1,419,791	0.0196
17	III	Right superior	M	43	Anaplastic astrocytoma	1,348,556	0.0168
18	III	Right middle	F	31	Anaplastic oligodendroglioma	1,397,215	0.0109
19	III	Right inferior	F	37	Astrocytoma	1,386,566	0.0284
20	IV	Right superior	M	31	Glioblastoma	1,463,844	0.0066

**Table 2:** Functional connectivity strength determined using a single-sample T test (P < 0.01 for the control group and P < 0.05 for the patient group) (mean ± std, 95% CI (confidence interval)).

Group	Item	lPCC-lTPJ	lPCC-rTPJ	rPCC-lTPJ	rPCC-rTPJ
Control	Mean ± Std	0.806 ± 0.147	0.735 ± 0.192	0.727 ± 0.129	0.797 ± 0.169
	CI	(0.738, 0.875)	(0.645, 0.824)	(0.667, 0.787)	(0.718, 0.876)
Patient	Mean ± Std	0.647 ± 0.133	0.636 ± 0.147	0.638 ± 0.150	0.668 ± 0.159
	CI	(0.585, 0.710)	(0.568, 0.705)	(0.568, 0.709)	(0.593, 0.743)

(See details in Charts 1 and 2 in the Appendix).

**Table 3:** Pearson correlation coefficients between pairs of FC strength values within the groups.

	Controls				
	lPCC-lTPJ	lPCC-rTPJ	rPCC-lTPJ	rPCC-rTPJ	
**lPCC-lTPJ**		0.788***	0.733***	0.716***	lPCC-lTPJ
**lPCC-rTPJ**	0.829***		0.636***	0.779***	lPCC-rTPJ
**rPCC-lTPJ**	0.886***	0.741***		0.639***	rPCC-lTPJ
**rPCC-rTPJ**	0.836***	0.931***	0.728***		rPCC-rTPJ
	**lPCC-lTPJ**	**lPCC-rTPJ**	**rPCC-lTPJ**	**rPCC-rTPJ**	
	**Patients**				

(See details in Charts 6 and 7 in the Appendix). ‘**’ indicates P < 0.05, and ‘***’ indicates P < 0.01.

**Table 4:** Correlation analysis between WHO grades and the functional connectivity pairs assessed in the sub-groups of patients with glioma located in the left or right hemisphere.

Items	lPCC-lTPJ	lPCC-rTPJ	rPCC-lTPJ	rPCC-rTPJ
Left glioma	−0.615 (0.033*)	−0.307 (0.331)	−0.307 (0.331)	−0.256 (0.422)
Right glioma	−0.056 (0.895)	−0.056 (0.896)	0.169 (0.689)	0.169 (0.689)

(Unit: Coefficient (P value)) ‘*’ indicates P < 0.05.

**Table 5:** Partial correlation analysis between WHO grades and functional connectivity pairs based on setting the volumes of normal cerebral tissue and tumor lesion as covariates, as assessed in the sub-groups of patients with tumors located in the left or right hemisphere.

Items	lPCC-lTPJ	lPCC-rTPJ	rPCC-lTPJ	rPCC-rTPJ
Left glioma	−0.300 (0.400)	0.075 (0.837)	−0.039 (0.331)	−0.069 (0.850)
Right glioma	0.442 (0.380)	0.157 (0.380)	0.658 (0.155)	0.380 (0.457)

(Unit: Coefficient (P value)) ‘*’ indicates P < 0.05.
