# Supplementary material for: Alteration of the Intra- and Cross- Hemisphere Posterior Default Mode Network in Frontal Lobe Glioma Patients
Source: Sci Rep. 2016 Jun 1;6:26972. doi: 10.1038/srep26972 (PMC4888650; doi:10.1038/srep26972)
Supplement: Supplementary Information [file srep26972-s1.pdf]

Alteration of the Intra- and Cross- Hemisphere Posterior Default Mode Network in Frontal Lobe Glioma Patients

APPENDIX PART

Haosu Zhang[1], Yonghong Shi[2, 3], Chengjun Yao[1], Weijun Tang[1], Demin Yao[2, 3], Chenxi Zhang[2, 3], Manning Wang[2, 3], Jinsong Wu[1, *], Zhijian Song[2, 3, *]

[1]Neurosurgery Department, Huashan Hospital, Shanghai Medical College, Fudan
[2]Digital Medical Research Center, School of Basic Medical Sciences, Fudan University
[3]Shanghai Key Laboratory of Medical Imaging Computing and Computer Assisted Intervention, Shanghai 200032

The appendix explicitly stated the details of the statistical analysis for explaining the kind of data to which the several *t*-tests have been applied.

Chart 1 Statistical description of the functional connectivity strength in the patient group.

Item		lPCC-lTPJ	lPCC-rTPJ	rPCC-lTPJ	rPCC-rTPJ
**Mean**		0.647	0.636	0.638	0.668
**95% confidence interval of mean**	**Lower limit**	0.585	0.568	0.568	0.593
	**Upper limit**	0.710	0.709	0.709	0.743
**Median**		0.616	0.639	0.618	0.705
**Standard deviation**		0.133	0.147	0.150	0.159
**Minimum**		0.401	0.409	0.350	0.374
**Maximum**		0.946	0.916	0.930	0.938
**Interquartile range**		0.146	0.200	0.211	0.230

Chart 2 Statistical description of the functional connectivity strength in the control group.

Item		lPCC-lTPJ	lPCC-rTPJ	rPCC-lTPJ	rPCC-rTPJ
**Mean**		0.806	0.735	0.727	0.797
**95% confidence interval of mean**	**Lower limit**	0.738	0.645	0.667	0.718
	**Upper limit**	0.875	0.824	0.787	0.876
**Median**		0.817	0.762	0.722	0.777
**Standard deviation**		0.147	0.192	0.129	0.169
**Minimum**		0.420	0.165	0.471	0.456
**Maximum**		1.041	1.073	0.937	1.076
**Interquartile range**		0.204	0.229	0.169	0.252

Chart 3A Description of the statistical parameter in the cross-group unpaired *T*-test in the patients and controls.

Item	Levene's test			*t*-test				
	Homogeneity of variance	*W*	P_W	*T*	Dof	P_t	95% CI	
							Lower limit	Upper limit
**lPCC-lTPJ**	assumed	0.067	0.797	3.582	38	**0.001*****	0.069	0.249
**lPCC-rTPJ**	assumed	0.215	0.646	1.816	38	0.077	-0.011	0.208
**rPCC-lTPJ**	assumed	0.472	0.496	2.003	38	0.052	-0.001	0.178
**rPCC-rTPJ**	assumed	0.034	0.854	2.489	38	**0.017****	0.024	0.234

'**' indicates $P_t < 0.05$, and '***' indicates $P_t < 0.01$

Note: paired or unpaired T-tests are used to analyze the relationship among lPCC-lTPJ, lPCC-rTPJ, rPCC-lTPJ, and rPCC-rTPJ in the intra- and cross- group, respectively. First, from the Levene's test, $P_W > 0.1$ shows that all pairs are homogeneity of variance. Here, The result of the Levene's test is ***W*** whose significance is tested against F-test distribution with 0.1 chosen as the level of significance, then P_W is the significant level of ***W***. Then, the *T*-test is applied to observe the significant difference between the intra- and cross- hemispheric functional connectivity for the control and patient group. here, ***T*** is the result of *T* -test, **Dof** is the degree of freedom and P_t is the significant level, and **CI** is the confidential interval.

Chart 3B Statistical description of the patients and controls.

Item		Number of samples	Mean	Standard deviation
**lPCC-lTPJ**	controls	20	0.806	0.147
	patients	20	0.647	0.133
**lPCC-rTPJ**	controls	20	0.735	0.192
	patients	20	0.636	0.147
**rPCC-lTPJ**	controls	20	0.727	0.129
	patients	20	0.638	0.150
**rPCC-rTPJ**	controls	20	0.797	0.169
	patients	20	0.668	0.159

Chart 4 Statistical description of the intra-group relationships among lPCC-lTPJ, lPCC-rTPJ, rPCC-lTPJ, and rPCC-rTPJ determined using a paired T-test (P < 0.05) for the patient group.

Item	Mean	Standard deviation	95% CI		*T*	Dof	P_t
			Lower limit	Upper limit			
**lPCC-lTPJ and lPCC-rTPJ**	0.011	0.083	-0.028	0.050	0.593	19	0.560
**lPCC-lTPJ and rPCC-lTPJ**	0.009	0.070	-0.024	0.042	0.576	19	0.572
**rPCC-lTPJ and rPCC-rTPJ**	-0.029	0.114	-0.083	0.024	-1.153	19	0.263
**lPCC-rTPJ and rPCC-rTPJ**	-0.032	0.058	-0.059	-0.004	-2.427	19	**0.025****

'**' indicates $P_t < 0.05$, and '***' indicates $P_t < 0.01$

Chart 5 Statistical description of the intra-group relationships among lPCC-lTPJ, lPCC-rTPJ, rPCC-lTPJ, and rPCC-rTPJ determined using a paired T-test (P < 0.05) for the control group.

Item	Mean	Standard deviation	95% CI		*T*	Dof	P_t
			Lower limit	Upper limit			
**lPCC-lTPJ and lPCC-rTPJ**	0.072	0.118	0.017	0.127	2.721	19	**0.014****
**lPCC-lTPJ and rPCC-lTPJ**	0.079	0.102	0.031	0.127	3.464	19	**0.003*****
**rPCC-lTPJ and rPCC-rTPJ**	-0.070	0.132	-0.132	-0.008	-2.379	19	**0.028****
**lPCC-rTPJ and rPCC-rTPJ**	-0.063	0.122	-0.120	-0.006	-2.300	19	**0.033****

'**' indicates $P_t < 0.05$, and '***' indicates $P_t < 0.01$

Chart 6 Statistical description of the Pearson correlation coefficients between pairs of functional connectivity strength within the patient group (here, the number of samples is 20).

	**lPCC-lTPJ**	**lPCC-rTPJ**	**rPCC-lTPJ**	**rPCC-rTPJ**
**lPCC-lTPJ**	1.000	0.829***	0.886***	0.836***
**lPCC-rTPJ**	0.829***	1.000	0.741***	0.931***
**rPCC-lTPJ**	0.886***	0.741***	1.000	0.728***
**rPCC-rTPJ**	0.836***	0.931***	0.728***	1.000

'**' denotes P < 0.05 and '***' denotes P < 0.01

Chart 7 Statistical description of the Pearson correlation coefficients between pairs of functional connectivity strength within the control group (here, the number of samples is 20).

	**lPCC-lTPJ**	**lPCC-rTPJ**	**rPCC-lTPJ**	**rPCC-rTPJ**
**lPCC-lTPJ**	1.000	0.788***	0.733***	0.716***
**lPCC-rTPJ**	0.788***	1.000	0.636***	0.779***
**rPCC-lTPJ**	0.733***	0.636**	1.000	0.639***
**rPCC-rTPJ**	0.716***	0.779***	0.639***	1.000

'**' denotes P < 0.05 and '***' denotes P < 0.01

Chart 8 Description of the statistical parameter in the cross-group unpaired *T*-test in the patients with left frontal gliomas and controls.

Item	Levene's test			*t*-test				
	Homogeneity of variance	*W*	P_W	*T*	Dof	P_t	95% CI	
							Lower limit	Upper limit
**lPCC-lTPJ**	assumed	0.465	0.501	2.879	30	**0.007*** **	0.041	0.244
**lPCC-rTPJ**	assumed	0.484	0.492	1.240	30	0.225	-0.051	0.210
**rPCC-lTPJ**	assumed	0.002	0.967	1.155	30	0.257	-0.042	0.150
**rPCC-rTPJ**	assumed	0.323	0.574	1.967	30	0.058	-0.005	0.240

'**' denotes $P_t < 0.05$ and '***' denotes $P_t < 0.01$

Chart 9 Statistical description in the intra-group paired analysis of the patients with left frontal gliomas.

Item	Mean	Standard deviation	95% CI		T	Dof	P_t
			Upper limit	Lower limit			
**lPCC-lTPJ and lPCC-rTPJ**	0.008	0.094	-0.052	0.068	0.310	11	0.762
**rPCC-lTPJ and rPCC-rTPJ**	-0.006	0.096	-0.067	0.054	-0.233	11	0.820
**lPCC-lTPJ and rPCC-lTPJ**	-0.009	0.057	-0.045	0.027	-0.553	11	0.591
**lPCC-rTPJ and rPCC-rTPJ**	-0.024	0.063	-0.064	0.016	-1.308	11	0.218

'**' denotes $P_t < 0.05$ and '***' denotes $P_t < 0.01$

Chart 10 Statistical description of the patients with left or right frontal gliomas.

Items		Mean	Number of samples	Standard deviation
**Patients with left frontal gliomas**	**lPCC-lTPJ**	0.664	12	0.113
	**rPCC-lTPJ**	0.673	12	0.129
	**lPCC-rTPJ**	0.655	12	0.141
	**rPCC-rTPJ**	0.679	12	0.155
**Patients with right frontal gliomas**	**lPCC-lTPJ**	0.623	8	0.164
	**rPCC-lTPJ**	0.587	8	0.174
	**lPCC-rTPJ**	0.608	8	0.161
	**rPCC-rTPJ**	0.651	8	0.174

Chart 11 Description of the statistical parameter in the cross-group unpaired *T*-test in the patients with right frontal gliomas and controls.

Item	Levene's test			*t*-test				
	Homogeneity of variance	*W*	P_W	*T*	Dof	P_t	95% CI	
							Lower limit	Upper limit
**lPCC-lTPJ**	assumed	0.082	0.777	2.889	26	**0.008*** **	0.053	0.314
**lPCC-rTPJ**	assumed	0.042	0.840	1.643	26	0.112	-0.032	0.285
**rPCC-lTPJ**	assumed	0.299	0.589	2.354	26	**0.026** **	0.018	0.263
**rPCC-rTPJ**	assumed	0.027	0.871	2.051	26	**0.050** **	0.000	0.293

'**' denotes $P_t < 0.05$ and '***' denotes $P_t < 0.01$

Chart 12 Description of the statistical parameter in the intra-group analysis of the patients with right frontal gliomas.

Items	Mean	Standard deviation	95% CI		T	Dof	P_t
			Upper limit	Lower limit			
**lPCC-lTPJ and lPCC-lTPJ**	0.015	0.068	-0.042	0.072	.615	7	0.558
**rPCC-lTPJ and rPCC-rTPJ**	-0.064	0.137	-0.179	0.051	-1.318	7	0.229
**lPCC-lTPJ and rPCC-lTPJ**	0.036	0.082	-0.033	0.105	1.243	7	0.254
**lPCC-rTPJ and rPCC-rTPJ**	-0.043	0.051	-0.085	0.000	-2.386	7	**0.048** **

'**' denotes $P_t < 0.05$
